# Reduction in Vegetable Intake Disparities With a Web-Based Nutrition Education Intervention Among Lower-Income Adults in Japan: Randomized Controlled Trial

**DOI:** 10.2196/jmir.8031

**Published:** 2017-11-24

**Authors:** Saki Nakamura, Takayo Inayama, Kazuhiro Harada, Takashi Arao

**Affiliations:** ^1^ Department of Health Promotion Sciences Graduate School of Human Health Sciences Tokyo Metropolitan University Tokyo Japan; ^2^ Research Fellow of Japan Society for the Promotion of Science Tokyo Japan; ^3^ Graduate School of Human Development and Environment Kobe University Hyogo Japan; ^4^ Faculty of Sport Sciences Waseda University Saitama Japan

**Keywords:** vegetable intake, adults, socioeconomic disadvantage, Web-based nutrition intervention, randomized controlled trial

## Abstract

**Background:**

No existing Web-based nutrition education interventions have been evaluated in light of socioeconomic status just in Japan.

**Objective:**

The aim was to investigate the effect of a Web-based intervention program on reducing vegetable intake disparities between low- and middle-income Japanese adults.

**Methods:**

In this randomized controlled trial, participants were assessed at three time points—baseline, postintervention (5 weeks later), and a follow-up after 3 months—from October 2015 to March 2016. We collected data via a Japanese online research service company from 8564 adults aged 30 to 59 years. Participants were stratified according to national population statistics for gender and age, and randomly selected. They were then randomly allocated into intervention (n=900) and control (n=600) groups such that both groups contained an equal number of individuals with low and middle income. The intervention program encouraged behavior change using behavioral theories and techniques tailored to their assumed stage of change. The outcome was vegetable intake servings per day (1 serving being approximately 70 g).

**Results:**

Out of 900 participants who started, 450 were from the middle income group (of which 386 or 85.7% completed the intervention), and 450 were from the low income group (of which 371 or 82.4% completed). In the intervention group, vegetable intake increased in the low-income participants from baseline to postintervention (0.42 servings, 95% CI 0.11-0.72). A two-way analysis of variance showed that low-income participants had significant main effects of group (η2=0.04, *P*=.01) and time (η2=0.01, *P*<.001), and a significant interaction (η2=0.01, *P*=.009). Middle-income participants also had a significant main effect of time (η2=0.01, *P*=.006) and a significant interaction (η2=0.01, *P*=.046).

**Conclusions:**

This Web-based nutritional education intervention could fill the vegetable intake gap between low- and middle-income adults in Japan, and is expected to prevent noncommunicable and lifestyle-related diseases. Further intervention program improvements are necessary to maintain and increase vegetable intake for other groups.

**Trial Registration:**

Current Controlled Trials (UMIN-ICDR): UMIN000019376; https://upload.umin.ac.jp/cgi-open-bin/ icdr_e/ctr_view.cgi?recptno=R000022404 (Archived by WebCite at http://www.webcitation.org/6u9wihBZU)

## Introduction

### Background

Reducing health disparities is important for public health promotion [1]. Disparities in food intake are known to occur among socioeconomically disadvantaged people [2-4]. Appropriate vegetable intake prevents cancer [5] and obesity [6], and reduces the risk of cardiovascular disease [7-9] and other lifestyle-related diseases. Despite this, individuals with low socioeconomic status (SES) tend to have low vegetable intake [10]. Thus, promoting vegetable intake in low-SES individuals to reduce health disparities is important globally.

### Nutritional and Dietary Problems in Japan

Japan has one of the highest levels of longevity in the world. However, recently, health disparities have been recognized as a social problem [11,12]. Health Japan 21 [13] recommends a vegetable intake of 350 g (5 servings) per day for adults to reduce health disparities related to lifestyle-related diseases. However, low-income people tend to consume few vegetables [10] (in the lowest income bracket: men 254 g per day, women 282 g per day). In a cross-sectional study of Japanese adults, a low percentage of individuals with lower annual income (<¥3,000,000, which was equivalent to approximately US $24,987 in October 2015) ate five servings (approximately 350 g) of vegetables daily: men 5.5% and women 10.4% [14]. Currently, practical strategies for reducing vegetable intake disparities are lacking and, therefore, are urgently needed.

### A Theory Suitable for Nutrition Education

A systematic review revealed that research has utilized multiple health behavior theories in attempting to increase vegetable intake, such as stages of change [15], social cognitive theory [16], the theory of planned behavior [17], and technology-based behavior change models [18,19]. Henry et al [20] suggested the possibility of increasing vegetable intake in low-income women by using a nutrition education intervention focusing on improving self-efficacy (perceived behavioral control) [21]. Thus, the gap in vegetable intake between low- and middle-income individuals might be reduced through a multicomponent nutrition education program that focuses on self-efficacy. It is therefore necessary to evaluate the nutrition education program developed in terms of whether it produces the intended outcome in evaluating nutrition education based on a multicomponent nutrition education program, not only outcome evaluation but also process evaluation, such as perceived behavioral control.

### Prior Work

There are some concerns about applying Web-based interventions to socioeconomically disadvantaged populations because they might have access only to poorer quality Internet environments. Nevertheless, Web-based interventions are generally easier to access, lower cost, and tend to be comfortable for most users. They similarly have advantages in being able to provide standardized information regardless of place or population size. These interventions have been drawing attention in recent years, with many studies confirming their efficacy in health promotion in adults [22-27]. For instance, Web-based interventions were able to increase vegetable intake in low-SES adults in rural America [28,29]. However, these studies did not examine reductions in vegetable intake disparities because they focused only on individuals with low SES.

### Objectives of This Study

Our study was designed to investigate reductions in vegetable intake disparities between low- and middle-income adults. We developed a Web-based nutrition education program that incorporates multiple health behavior theories to promote vegetable intake [30]. The aim was to investigate the effects of this program on the vegetable intake and patterns of change in vegetable intake of low- and middle-income adults in Japan.

## Methods

### Trial Design and Ethics

We previously reported the details of the nutrition education program in a study protocol [30]. This study was a matched-design, randomized controlled trial (RCT). Participants were assessed by self-report at three time points: baseline, postintervention, and follow-up at 3 months. The study period ranged from October 2015 to March 2016. We obtained baseline data in October 2015 and postintervention data in December 2015; the follow-up period was March 2016 (ie, 3 months after postintervention). All intervention group participants completed the intervention in the same 5-week period. All control group participants completed the survey at all three time points, but did not undergo the intervention program. The RCT was approved by the Ethics Review Committee on Research with Human Subjects of Waseda University, Japan (2015-167), and Current Controlled Trials (UMIN-ICDR UMIN000019376).

### Participants and Recruitment

**Figure 1:**
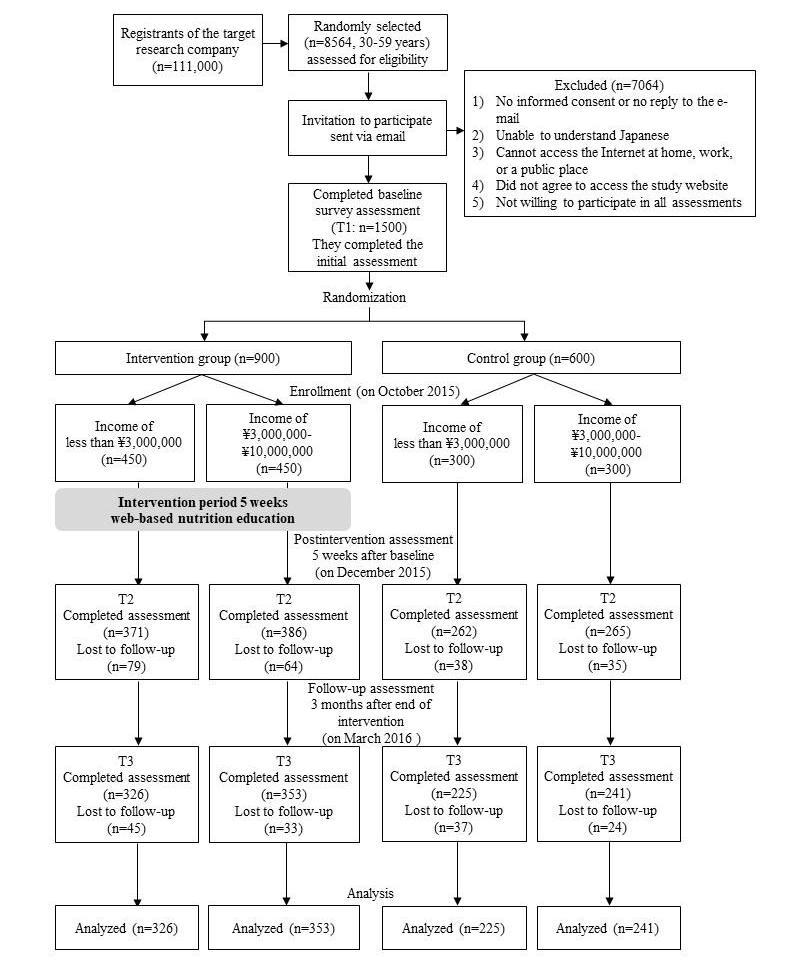
Flowchart showing participant recruitment, randomization, and evaluation of the Diet and Exercise Practices Project study.

Figure 1 shows the study participant recruitment and flow. A Japanese online research service company containing data from approximately 111,000 people (as of September 2015) conducted the survey at all three time points (baseline to follow-up). The research service company randomly selected 8564 adults aged 30 to 59 years to match the gender and age [31] distributions of Japan at baseline. We targeted adults aged 30 to 59 years because we felt that both the promotion of healthy eating and reduction in health disparities were particularly important in this group. In the past, we carried out a cross-sectional study on the relationship between socioeconomic status and dietary habits in this age group [14,30,32,33]. If participants met any of the exclusion criteria, they were not sent an email. Therefore, it is unknown why participants were excluded. The exclusion criterion were an annual income of more than ¥10,000,000 (this was equivalent to approximately US $83,333, in October 2015, US $1 was equivalent to approximately ¥120; 88.4% of the total population has an income of less than ¥10,000,000).

Recruitment was terminated when the number of participants who agreed to participate reached 1500. The research service company randomly divided participants into intervention and control groups, and collected data via computer. The authors were blinded to the randomization. Participants received a detailed explanation of the research because of ethical considerations and were informed that they had been randomly assigned to their group. However, because participants did not obtain any information about the other participants, we believe that there was no contamination bias. The details of the incentives of this research are described in the study protocol [30].

The sample size was calculated using an effect size of .5, an alpha of .05, and power of .95 [30]. Among participants with incomes of less than ¥3,000,000 and those with incomes of ¥3,000,000 to ¥10,000,000, allocation was as follows: n=450 (intervention) and n=300 (control). Most adults in Japan have incomes of ¥2,000,000 to ¥3,000,000, accounting for one-third of the Japanese population [34]. Our previous survey showed that the percentage of people eating 350 g (5 servings) of vegetables daily among individuals earning less than ¥3,000,000 was less (men: 5.5%; women: 10.4%) compared to those earning more than ¥3,000,000 [14]. Therefore, ¥3,000,000 was used as the relative cutoff point. Because most of the total population earn less than ¥10,000,000, this upper limit was set in consideration of ceiling effects [34]. The size of the control group was set at 600 participants; the expected dropout rate was approximately 50% according to the research service company during the survey period. The size of the intervention group was set at 900 participants, with an expected dropout rate of two-thirds. We also referred to the dropout percentage (15.3%) in Kothe et al [26] (the intervention period—30 days—was about the same as ours).

### Procedure

The intervention group received emails (approximately 200 words in Japanese) with health information once a week on Monday between 6:00 am and 7:00 am. The email contained the following information: “website update announcement,” “previous overview,” “this summary,” and “how to proceed with the site.” For example, for step 2 of the intervention (which took place on the second week of the intervention), the email contained the following information:

Hello, let us look back on your own eating habits is the first step toward health promotion. Step 2 has been updated so we will contact you. i) Diet: Review of Step 1 “How many vegetables dishes (servings) did you eat per day? Let’s self-check and see” ii) Diet: Contents of Step 2 “Let’s choose one more vegetable dish,” iii) Please see 4 pages of each step in this order, 1) Today’s points → 2) Do you know? → 3) Easy to devise → 4) Let’s try it! Please look for evident information and let’s choose what you can do.

After completion of the 5-week intervention, participants received an email about the postintervention survey. Finally, participants received an email about the 3-month follow-up survey.

### Intervention Program

An interactive website called the “Diet and Exercise Practices Project” [35] was developed. This is a free website that provides information, three monitoring sheets, and advice about healthy diets, increasing vegetable intake, and preventing lifestyle-related diseases. We hypothesized that achieving an approximately 70 g (1 serving) increase in vegetable intake might help lower-income adults “catch up” in intake compared to middle-income groups, while simultaneously contributing to the partial resolution of the overall deficient vegetable intake in Japanese adults.

**Figure 2:**
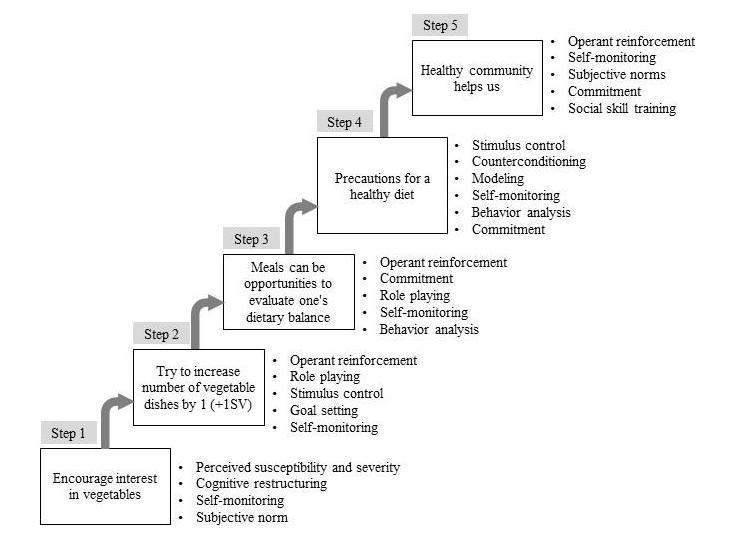
Five steps and behavioral modification techniques of the nutrition education intervention program. The Web intervention period was 5 weeks.

The program consisted of a total of 20 pages of content, divided into five steps (one step contained four pages). The webpage was updated with one step every week. The program was based on the transtheoretical model (TTM) (Figure 2). Details of the program’s theoretical framework are reported in a previously published study protocol [30]. In step 1, we used the health belief model to encourage movement from the precontemplative to the contemplative phase. In steps 2 and 3, social cognitive theory and the theory of planned behavior were used to encourage movement from the contemplative to the preparation phase. In step 4, social cognitive theory and the theory of planned behavior were again used, but this time to encourage movement from the preparation to the action phase. Finally, in step 5, strengthening of social networks and social support were used to promote a shift to the maintenance phase.

**Figure 3:**
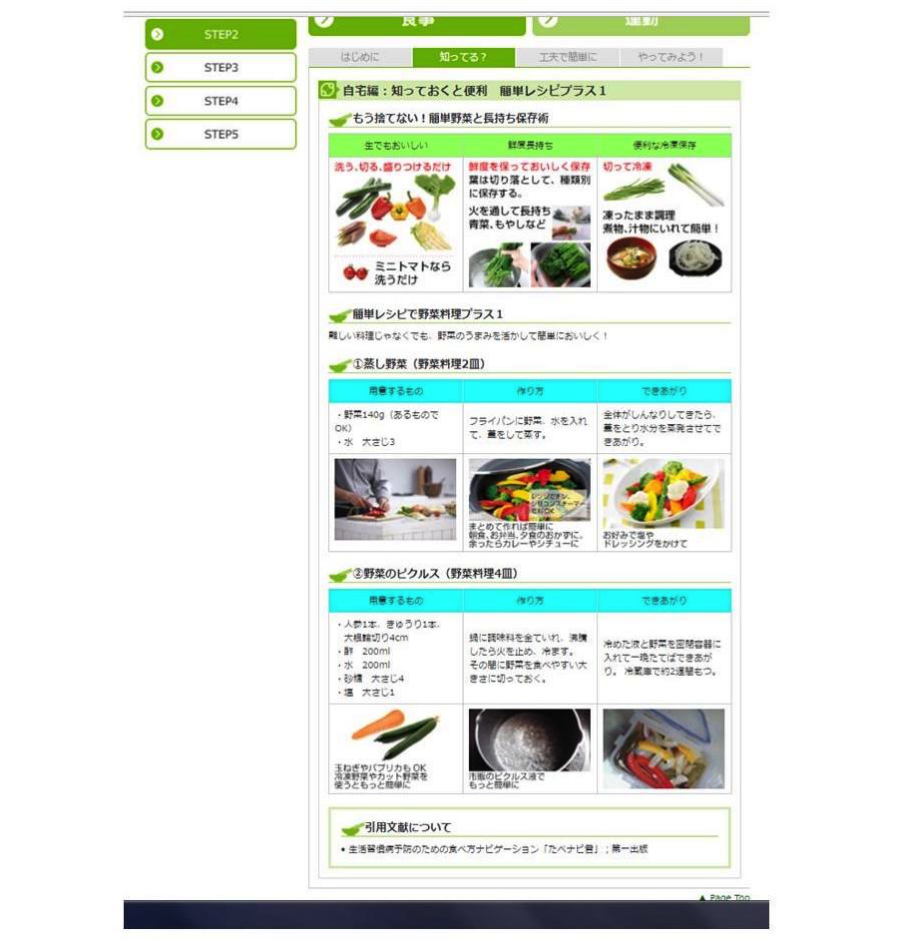
Example of content of the Web-based nutrition intervention program (1 page).

The four pages in each step were structured as follows: (1) “Today’s point” (including a review of the previous week from the second week onward), which served as practical content; (2) “Do you know?” and (3) “Easy to devise,” which were summaries; and (4) “Let’s try it!” which involved supporting behavior change by using a worksheet. Figure 3 shows an example of the content on one page (ie, page 2 for step 2).

The control group surveys took place over the same period as the intervention group surveys. Control group participants received an email from the survey company informing them that they had been randomly assigned to a control group after the baseline. After a 5-week interval, participants received an email requesting them to take part in the postintervention survey. Three months later, the participants received an email requesting them to participate in a follow-up survey.

**Table 1:** Questionnaires and answers on vegetable intake at three time points.

Variable and questionnaire		Answer category
**Vegetable intake**		
	How many vegetable dishes do you usually eat (dishes with vegetables as the main ingredient) per day? One dish is about one small bowl (70 g)	Number of dishes (servings/day)
**Vegetable eating behavior (per week)^a^**		
	The following questions are about your normal dietary habits. Do you eat ample amounts of vegetables (5 small bowls/day, about 350 g)?	1=Almost every day; 2=4-5 days/week; 3=2-3 days/week; 4=almost none
**Transtheoretical model^a^**		
	Which of the following matches your current dietary condition? Do you eat ample amounts of vegetables (5 small bowls/day, about 350 g)?	1=Maintenance (I have continued to eat them for more than 6 months); 2=action (I have continued to eat them for less than 6 months); 3=preparation (I sometimes eat or intend to eat within the next 30 days); 4=contemplation (although I do not currently eat them, I intend to start eating them within the next 6 months); 5=precontemplation (I do not eat them and I do not intend to start eating them within the next 6 months)
**Perceived behavioral control^a^**		
	Do you believe you can do the following things to maintain your health, and your future health, with confidence? Do you have confidence in eating adequate amounts of vegetables (5 small dishes/day, or about 350 g)?	1=A lot of confidence (I have a lot of confidence in eating); 2=quite a lot of confidence (I have quite a lot of confidence in eating); 3=a little confidence (I have a little confidence in eating); 4=not a lot of confidence (I do not have a lot of confidence in eating); 5=very little confidence (I have very little confidence in eating); 6=not have any confidence (I do not have any confidence in eating)
**Knowledge^a^**		
	Did you know that the recommended vegetable intake for maintaining health in adults is 350 g per day?	1=Yes; 2=no

^a^Participants chose one answer that best applied to them.

### Data Collection and Outcome Measures

Participants were assessed via self-report at three time points: baseline, postintervention, and follow-up at 3 months. We have listed the details of the assessment items in Table 1. This study evaluated vegetable intake as the main outcome to assess the effectiveness of the nutrition education program. Ozawa et al [36] suggested that the number of vegetable dishes consumed may be a simpler and more valid measure of vegetable intake compared to a dietary record for both men and women. We presented participants with photographic examples of vegetable dishes (including the size and weight) before they answered the questionnaire. We referred to “The Japanese Food Guide Spinning Top” [37], wherein a dish where vegetables were the main ingredients (70 g) represented one serving.

Moreover, we performed a process evaluation of behavior change using various other outcomes, including vegetable eating behavior (per week) [38], stages of change, perceived behavioral control, and knowledge [39]. We used perceived behavioral control because it is an important concept [40] in behavior change. For the knowledge item, we showed photographic examples of vegetable dishes (including size and weight) before participants answered. Demographic variables included sex, age, marital status, number of people at home, employment status, and educational status.

### Statistical Analyses

We compared the groups in terms of baseline sociodemographic characteristics using chi-square and Mann-Whitney *U* tests. Moreover, differences in baseline vegetable intake between participants and dropouts were assessed using unpaired *t* tests and one-way analyses of variance (ANOVAs). Amount of change in vegetable intake was analyzed using a general linear model. The mean change in vegetable intake was analyzed using Bonferroni-corrected comparisons following one-way ANOVAs for the different combinations of groups and time points. We compared the intervention effect on vegetable intake by group and time using two-way ANOVAs, and calculated the effect sizes (η^2^). Other outcomes concerning vegetables were tested using McNemar test and the Wilcoxon signed rank test. The effects of multiple comparison were adjusted for using Bonferroni corrections. Participants lost to follow-up, that is those who did not complete the postintervention (n=216) or follow-up (n=139) surveys or who were otherwise missing any outcome data, were excluded from the analyses. This resulted in the exclusion of 355 of 1500 participants (23.67% of those randomly assigned) at baseline. Such an approach is in line with the revised CONSORT guidelines [41], as there are criticisms of and potential bias caused by imputing missing outcome data required for an intention-to-treat analysis. It has been pointed out that when the dropout rate is high, researchers should be cautious about conducting an intention-to-treat analysis. Indeed, in another RCT [42], an intention-to-treat analysis was not carried out because of a high dropout rate (14.9%). Therefore, our analyses were not strictly intention to treat. All statistical analyses were performed using IBM SPSS Statistics version 21.0. A *P* value of .05 was used as the level of significance.

## Results

### Baseline Data

Table 2 shows baseline data collected from 1145 participants. The number of participants who completed the intervention in the intervention group (n=679) was as follows: low income (n=326, 72.4%) and middle income (n=353, 78.4%). In the control group (n=466), the number of participants who completed all three surveys was as follows: low income (n=225, 75.0%) and middle income (n=241, 80.3%). There were no differences in characteristics between the intervention group and the control group in either income group, except for marital status and number of people at home in the <¥3,000,000 group. Comparison of baseline characteristics between participants who were excluded and those who were included yielded the following differences: gender (included: men 596/1145, 52.1%, women 549/1145, 47.9%; excluded: men 154/355, 43.4%, women 201/355, 56.6%; *P*=.005) and educational status (included: junior high/high school 304/1134, 26.8%, 2-year college 297/1134, 26.2%, 4-year college/graduate school 533/1134, 47.0%; excluded: junior high/high school 114/348 32.8%, 2-year college 99/1134 28.4%, 4-year college/graduate school 135/1134, 38.8%; *P*=.005).

**Table 2:** Baseline sociodemographic characteristics of the study participants by income level (N=1145).

Variable		<¥3,000,000^a^			¥3,000,000-¥10,000,000^a^		
		Intervention (n=326) n (%)	Control (n=225) n (%)	*P*	Intervention (n=353) n (%)	Control (n=241) n (%)	*P*
**Gender^b^**				.80			.93
	Men	169 (51.8)	120 (53.3)		183 (51.8)	124 (51.5)	
	Women	157 (48.2)	105 (46.7)		170 (48.2)	117 (48.5)	
**Age (years)^c^**				.39			.23
	30-39	106 (32.5)	76 (33.8)		117 (33.1)	73 (30.3)	
	40-49	116 (35.6)	88 (39.1)		138 (39.1)	89 (36.9)	
	50-59	104 (31.9)	61 (27.1)		98 (27.8)	79 (32.8)	
**Marital status^b^**				.04			.47
	Not married	217 (66.6)	169 (75.1)		116 (32.9)	72 (29.9)	
	Married	109 (33.4)	56 (24.9)		237 (67.1)	169 (70.1)	
**Number of people at home^b,d^**				.03			.35
	≥2	198 (66.4)	111 (56.6)		292 (85.6)	194 (82.6)	
	1	100 (33.6)	85 (43.4)		49 (14.4)	41 (17.4)	
**Employment status^b,d^**				.39			.60
	Not employed	103 (32.5)	61 (28.6)		74 (21.4)	46 (19.2)	
	Employed	214 (67.5)	152 (71.4)		272 (78.6)	193 (80.8)	
**Educational status^c,d^**				.06			.91
	Junior high/high school	114 (35.5)	72 (32.3)		71 (20.3)	47 (19.6)
	2-year college	92 (28.7)	47 (21.1)		93 (26.6)	65 (27.1)	
	4-year college/graduate school	115 (35.8)	104 (46.6)		186 (53.1)	128 (53.3)	

^a^¥120=US $1 (October 2015).

^b^Chi-square test.

^c^Mann-Whitney *U* test.

^d^Percentage excludes unknown/other answers. In <¥3,000,000: number of people at home (n=28), employment status (n=9), educational status (n=5) in the intervention group; number of people at home (n=29), employment status (n=12), educational status (n=2) in the control group. In ¥3,000,000-¥10,000,000: number of people at home (n=12), employment status (n=7), educational status (n=3) in the intervention group; number of people at home (n=6), employment status (n=2), educational status (n=1) in the control group.

### Outcomes

#### Means and Mean Differences in Vegetable Intake at Each Time Point

Table 3 shows the mean (SD) vegetable intake at each time point. The participants with low income at baseline in the intervention group showed a lower vegetable intake compared with middle-income participants in both the intervention and control groups. The same pattern was found for participants with low income at baseline in the control group. There were no other differences between the groups. We confirmed that there were no differences in baseline vegetable intake between participants who were included in the analysis and those who dropped out (*P*=.91). The mean difference in vegetable intake at postintervention also increased in low-income intervention group participants compared to baseline (mean 0.42 servings, 95% CI 0.11 to 0.72, *P*<.001). In the control group among low-income participants, the mean vegetable intake at postintervention was not much different from that at baseline (mean 0.05 servings, 95% CI –0.26 to 0.36); the difference between follow-up and baseline was also minor (mean 0.03 servings, 95% CI –0.28 to 0.34). For middle-income participants, the mean vegetable intake at postintervention was barely different from that baseline (mean 0.04 servings, 95% CI –0.27 to 0.36); the same was true comparing follow-up and baseline (mean 0.03 servings, 95% CI –0.29 to 0.34).

#### Effect Size of Vegetable Intake

**Figure 4:**
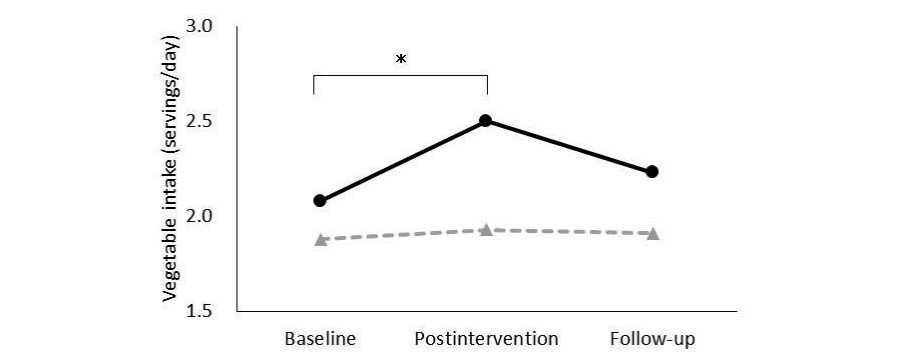
In the <¥3,000,000 group, means of self-reported vegetable intake at baseline, postintervention, and follow-up at 3 months in the intervention group (solid line) and control group (dotted line). Data were analyzed by using two-way ANOVAs. *General linear model, significance was based on P <.05/3=.02 (Bonferroni-corrected).

**Figure 5:**
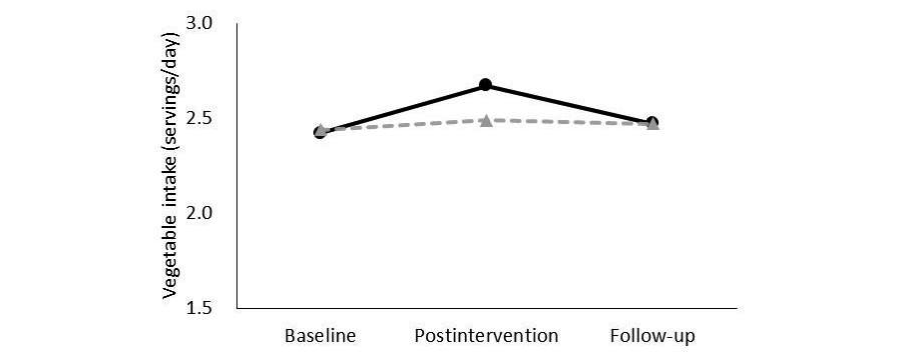
In the ¥3,000,000-¥10,000,000 group, means of self-reported vegetable intake at baseline, postintervention, and follow-up at 3 months in the intervention group (solid line) and the control group (dotted line). Data were analyzed by using two-way ANOVAs.

Table 4 shows the effect size of vegetable intake by income. Two-way ANOVAs showed that both low- and middle-income participants had significant main effects of group and time, and a significant interaction. Multiple comparisons (Figure 4) showed that vegetable intake among low-income participants increased between baseline and postintervention. Although it did not decrease significantly between postintervention and follow-up, the difference between baseline and follow-up was not significant. There were no changes in vegetable intake among middle-income participants when comparing any time point (Figure 5). However, multiple comparisons revealed that vegetable intake among low-income participants at baseline was lower than that among middle-income participants (baseline: *P*=.003). At postintervention and follow-up, the difference between income groups had disappeared (postintervention: *P*=.16; follow-up: *P*=.045).

**Table 3:** Mean (SD) and mean difference (95% CI) in each vegetable intake measure at the three time points.

Time	<¥3,000,000^a^ (servings/day)				¥3,000,000-¥10,000,000^a^ (servings/day)			
	Intervention (n=326) mean (SD)^b^	Control (n=225) mean (SD)^b^	Mean difference (95% CI)^c^	*P*	Intervention (n=353) mean (SD)^b^	Control (n=241) mean (SD)^b^	Mean difference (95% CI)^c^	*P*
Baseline	2.08 (1.49)	1.88 (1.38)			2.42 (1.50)	2.44 (1.40)		
Postintervention	2.50 (1.79)	1.93 (1.37)	0.42 (0.11, 0.72)	<.003	2.67 (1.46)	2.49 (1.49)	0.25 (–0.02, 0.52)	.08
Follow-up	2.23 (1.54)	1.91 (1.33)	0.15 (–0.15, 0.46)	.67	2.47 (1.46)	2.47 (1.44)	0.04 (–0.23, 0.31)	>.99

^a^¥120=US $1 (October 2015).

^b^Mean (SD) of servings/day. General linear model. One-way ANOVA *P*<.05, Bonferroni-corrected post hoc comparisons using *t* test. Significance was based on *P*<.05/6=.008.

^c^Mean difference (95% CI) in servings/day in intervention group from baseline.

**Table 4:** The effect size (η^2^)^a^ in comparisons of vegetable intake between groups and times using two-way repeated-measures ANOVAs^b^.

Items	<¥3,000,000^c^			¥3,000,000-¥10,000,000^c^		
	Partial η^2^	η^2^	*P*	Partial η^2^	η^2^	*P*
Group	0.10	0.04	.03	0.00	0.00	.62
Time	0.01	0.01	.02	0.01	0.01	.006
Group*time	0.10	0.01	.009	0.01	0.01	.046

^a^Effect size (low: η^2^=0.01; middle: η^2^=0.06; high: η^2^=0.14).

^b^General linear model adjusted for baseline marital status and number of people at home in <¥3,000,000. Dependent variable: vegetable intake servings.

^c^In <¥3,000,000 group, self-reported vegetable intake at all three time points: n=326 (intervention) and n=225 (control). In ¥3,000,000-¥10,000,000 group, self-reported vegetable intake at all three time points: n=353 (intervention) and n=241 (control). ¥120=US $1 (October 2015).

#### Results for Other Vegetable Intake Variables

Tables 5 and 6 show the results for eating vegetables behavior, stage of change, perceived behavioral control, and knowledge of vegetable intake. Low-income participants (<¥3,000,000) in the intervention group showed improvements in eating vegetables, stages of change, perceived behavioral control, and knowledge at postintervention compared to baseline. Furthermore, the improvements in eating vegetables and dietary knowledge were maintained between postintervention and follow-up. In middle-income participants (¥3,000,000-¥10,000,000), only improvements in knowledge were maintained from baseline to postintervention, and from baseline to follow-up. However, in the control group, improvements in knowledge were maintained from baseline to postintervention and from baseline to follow-up among both income groups (all *P*<.001).

**Table 5:** Baseline (T1), postintervention (T2), and follow-up (T3) in change in behavior, transtheoretical model, perceived behavioral control, and knowledge of vegetable intake as a result of Web-based intervention among adults with an income of <¥3,000,000.^a^

Variable		Intervention (n=326)					Control (n=225)				
		T1 n (%)	T2 n (%)	T3 n (%)	T1-T2 *P*^b^	T1-T3 *P*^b^	T1 n (%)	T2 n (%)	T3 n (%)	T1-T2 *P*^b^	T1-T3 *P*^b^
**Eating vegetable behavior^c^**					<.001	.008				.13	.005
	Almost every day	24 (7.4)	39 (12.0)	32 (9.8)			14 (6.2)	14 (6.2)	17 (7.6)		
	4-5 days/week	36 (11.0)	52 (16.0)	38 (11.7)			18 (8.0)	21 (9.3)	30 (13.3)		
	2-3 days/week	91 (27.9)	97 (29.8)	109 (33.4)			75 (33.3)	79 (35.1)	61 (27.1)		
	Almost none	175 (53.7)	138 (42.3)	147 (45.1)			118 (52.4)	111 (49.3)	117 (52.0)		
**Transtheoretical model^d^**					<.001	.27				.10	.29
	Maintenance	56 (17.2)	74 (22.7)	65 (19.9)			27 (12.0)	34 (15.1)	40 (17.8)		
	Action	17 (5.2)	16 (4.9)	19 (5.8)			10 (4.4)	19 (8.4)	9 (4.0)		
	Preparation	108 (33.1)	123 (37.7)	100 (30.7)			82 (36.4)	62 (27.6)	64 (28.4)		
	Contemplation	84 (25.8)	75 (23.0)	83 (25.5)			62 (27.6)	68 (30.2)	69 (30.7)		
	Precontemplation	61 (18.7)	38 (11.7)	59 (18.1)			44 (19.6)	42 (18.7)	43 (19.1)		
**Perceived behavioral control^c^**					<.001	.06				.32	.25
	A lot of confidence	15 (4.6)	24 (7.4)	16 (4.9)			10 (4.4)	9 (4.0)	8 (3.6)		
	Quite a lot of confidence	30 (9.2)	33 (10.1)	38 (11.7)			10 (4.4)	18 (8.0)	23 (10.2)		
	A little confidence	49 (15.0)	56 (17.2)	54 (16.6)			33 (14.7)	32 (14.2)	33 (14.7)		
	Not a lot of confidence	108 (33.1)	118 (36.2)	109 (33.4)			78 (34.7)	72 (32.0)	75 (33.3)		
	Very little confidence	51 (15.6)	42 (12.9)	41 (12.6)			40 (17.8)	40 (17.8)	29 (12.9)		
	Not any confidence	73 (22.4)	53 (16.3)	68 (20.9)			54 (24.0)	54 (24.0)	57 (25.3)		
**Knowledge**					<.001	<.001				<.001	<.001
	Yes	106 (32.5)	174 (53.4)	177 (54.3)			60 (26.7)	90 (40.0)	93 (41.3)		
	No	220 (67.5)	152 (46.6)	149 (45.7)			165 (73.3)	135 (60.0)	132 (58.7)		

^a^¥120=US $1 (October 2015).

^b^Ordinal scale: Wilcoxon signed rank test, Bonferroni-corrected post hoc comparisons made using Mann-Whitney *U* test (*P*<.05/3=.02). Nominal scale: McNemar test, Bonferroni-corrected post hoc comparisons made using chi-square test (*P*<.05/3=.02).

^c^The percentage might not reach 100% in some cases because the rate was rounded off.

^d^Transtheoretical model (TTM) 5 stages of change: (1) maintenance (I have continued to eat them for more than 6 months); (2) action (I have continued to eat them for less than 6 months); (3) preparation (I sometimes eat them or intend to eat them within the next 30 days); (4) contemplation (although I do not eat them currently, I intend to start eating them within the next 6 months); (5) precontemplation (I do not eat them and I do not intend to start eating them within the next 6 months).

**Table 6:** Baseline (T1), postintervention (T2), and follow-up (T3) in change in behavior, transtheoretical model, perceived behavioral control, and knowledge about vegetable intake in this Web-based intervention among adults with an income of ¥3,000,000-¥10,000,000.^a^

Variable		Intervention (n=353)					Control (n=241)				
		T1 n (%)	T2 n (%)	T3 n (%)	T1-T2 *P*^b^	T1-T3 *P*^b^	T1 n (%)	T2 n (%)	T3 n (%)	T1-T2 *P*^b^	T1-T3 *P*^b^
**Eating vegetable behavior^c^**					.38	.12				.03	.02
	Almost every day	32 (9.1)	33 (9.3)	34 (9.6)			19 (7.9)	19 (7.9)	18 (7.5)		
	4-5 days/week	50 (14.2)	56 (15.9)	73 (20.7)			38 (15.8)	44 (18.3)	48 (19.9)		
	2-3 days/week	125 (35.4)	134 (38.0)	120 (34.0)			86 (35.7)	103 (42.7)	100 (41.5)		
	Almost none	146 (41.4)	130 (36.8)	126 (35.7)			98 (40.7)	75 (31.1)	75 (31.1)		
**Transtheoretical model^c,d^**					.25	.14				.07	.24
	Maintenance	87 (24.6)	81 (22.9)	77 (21.8)			49 (20.3)	64 (26.6)	52 (21.6)		
	Action	23 (6.5)	24 (6.8)	42 (11.9)			12 (5.0)	15 (6.2)	21 (8.7)		
	Preparation	106 (30.0)	149 (42.2)	128 (36.3)			96 (39.8)	84 (34.9)	91 (37.8)		
	Contemplation	94 (26.6)	72 (20.4)	64 (18.1)			61 (25.3)	48 (19.9)	54 (22.4)		
	Precontemplation	43 (12.2)	27 (7.6)	42 (11.9)			23 (9.5)	30 (12.4)	23 (9.5)		
**Perceived behavioral control^c^**					.58	.15				.04	.95
	A lot of confidence	19 (5.4)	26 (7.4)	19 (5.4)			13 (5.4)	18 (7.5)	8 (3.3)		
	Quite a lot of confidence	53 (15.0)	44 (12.5)	47 (13.3)			33 (13.7)	36 (14.9)	31 (12.9)		
	A little confidence	71 (20.1)	71 (20.1)	64 (18.1)			45 (18.7)	39 (16.2)	47 (19.5)		
	Not a lot of confidence	122 (34.6)	132 (37.4)	135 (38.2)			81 (33.6)	94 (39.0)	100 (41.5)		
	Very little confidence	48 (13.6)	47 (13.3)	43 (12.2)			45 (18.7)	36 (14.9)	33 (13.7)		
	Not any confidence	40 (11.3)	33 (9.3)	45 (12.7)			24 (10.0)	18 (7.5)	22 (9.1)		
**Knowledge**					<.001	<.001				<.001	<.001
	Yes	109 (30.9)	186 (52.7)	191 (54.1)			69 (28.6)	90 (37.3)	110 (45.6)		
	No	244 (69.1)	167 (47.3)	162 (45.9)			172 (71.4)	151 (62.7)	131 (54.4)		

^a^¥120=US $1 (October 2015).

^b^Ordinal scale: Wilcoxon signed rank test, Bonferroni-corrected post hoc comparisons made using Mann-Whitney *U* test (*P*<.05/3=.02). Nominal scale: McNemar test, Bonferroni-corrected post hoc comparisons made using chi-square test (*P*<.05/3=.02).

^c^The percentage might not reach 100% in some cases because the rate was rounded off.

^d^Transtheoretical model (TTM) 5 stages of change: (1) maintenance (I have continued to eat them for more than 6 months); (2) action (I have continued to eat them for less than 6 months); (3) preparation (I sometimes eat them or intend to eat them within the next 30 days); (4) contemplation (although I do not eat them currently, I intend to start eating them within the next 6 months); (5) precontemplation (I do not eat them and I do not intend to start eating them within the next 6 months).

## Discussion

### Principal Results

A strength of this study was its RCT design and stratification by income to investigate the reduction in vegetable intake disparity. The main finding was that vegetable intake and related processes among low-income participants improved, thus reducing the existing disparities with the middle-income group. We suggest that this Web-based nutrition education program based on multiple health behavior theories is an effective intervention for low-income adults. To our knowledge, this is the first Web-based intervention study to investigate reductions in vegetable intake disparities in adults. Further improvements in the intervention program are necessary to increase intake and maintain that increase throughout a follow-up period among middle-income adults.

The vegetable intake among low-income participants increased by mean 0.42 servings (95% CI 0.11-0.72) after the intervention, which helped reduce the vegetable intake disparity between incomes. Additionally, behavioral change processes such as dietary behavior, stages of change, self-efficacy, and knowledge improved. Most past Web-based studies were conducted outside Japan. Bensley et al [24] reported an increase of 0.59 servings after a Web-based nutrition education for 6 months with adults, Sternfeld et al [23] an increase of 0.18 servings using an email intervention for 16 weeks with employees, and Kothe et al [26] an increase of 0.84 servings using an email intervention for 30 days with undergraduate students. Thus, in all three of these studies, the intervention led to increased fruit and vegetable intake. However, these studies evaluated vegetables and fruit within the same category. The Japanese Food Guide Spinning Top, a Japanese food guide, classifies vegetables and fruits in different categories [37]. This study showed a clearly positive intervention effect for vegetable intake only. Past studies in Japan showed that nutrition education interventions increased vegetable intake by 0.32 servings in 24 weeks among male workers [43], and by 0.30 servings 1 year later in employees [44]. The improvements in vegetable intake and improved behavioral change processes are further strengths of this study. Our results contributed to reducing disparities in vegetable intake between low- and middle-income individuals by using a very short-term (5-week) Web-based intervention.

The nutrition education program also has two important strengths. First, the program was based on the stages of change, which are thought to be applicable to nutrition interventions [45]. Many previous studies have found support for methods based on multiple health behavior theories aimed at increasing vegetable intake [46,47]. In this study, participants were assumed to be in the precontemplation stage baseline (Figure 2). Because improved self-efficacy is essential for behavior change, we made sure that all steps of the program focused on improving self-efficacy. We expect that the composition of this program helped increase vegetable intake among low-income individuals, who may have had low self-efficacy (perceived behavioral control).

Second, Park et al [22] reported that 88% of participants completed their 30-day Web intervention for adults. Kothe et al reported 85% [26] and 80% [27] of participants completed the intervention with a 30-day email intervention for undergraduate students. This study had a roughly equivalent number of participants who completed the intervention (low income: 82.4%; middle income: 85.7% at postintervention). This is possibly because participants received an email including a weekly summary of the program and informative support. Additionally, the intervention was highly accessible (eg, time, place, situation) because participants could complete the activities using their mobile phone or personal computer. There are extremely few previous reports on Web-based interventions for Japanese adults [48]; as such, our Web-based nutrition education program is not only highly effective, but also provides novel evidence.

The improvement in vegetable intake for low-income participants in the intervention group disappeared by the 3-month follow-up. This might have been because we used only one theoretical approach (see step 5) to promote the change from the action to maintenance stage. Therefore, it is necessary to strengthen this aspect of the intervention. For example, Japanese traditional food culture distinguishes between seasonal dishes and foods for all four seasons. We could distribute nutrition information and recipes about seasonal foods such as vegetables during a follow-up period to promote continued vegetable intake. In addition, we could regularly tweet reviews of the program content, and send reminders of the effort needed to prevent reversal of behavior change. Behavior change can be regarded as habitual if it is maintained for more than 6 months. After observing the program-related improvements in this study, it is worth attempting these approaches during follow-up to maintain the intervention effect.

Vegetable intake among middle-income participants might not have increased after the intervention because of the program’s use of an inappropriate approach to behavior change for this income group. For example, food access and perceptions of the food environment might differ depending on income [49]. Perceived behavioral control, which was the focus of this program [30], has been found to be low among low-income women [20]. Thus, a program focusing on perceived behavioral control might have promoted ingestion of vegetables up to a certain amount. For further improvement, it is necessary to identify the factors affecting vegetable intake according to income and develop more appropriate intervention methods.

The reason for the low effect sizes was probably the smaller variety of content and shorter intervention periods than in previous studies. The effect size of a 30-day nutrition education intervention by email in undergraduate students [26] was roughly the same as in this study. However, Alexander et al [25] found medium effect sizes when using a website targeting adults. Their program had a rich variety of content, such as food education using a short video and audio files, and presented 300 fruit- and vegetable-based recipes. Furthermore, the intervention period was one year, which was considerably longer than was ours. The content of our program is a website of approximately 20 pages containing information and worksheets combining text and images. We expect larger effects if we increased the variety of the content and the length of the intervention period.

### Limitations

Some limitations warrant discussion. First, we were careful to extract samples matching the Japanese demographic distribution. Nonetheless, our participants had a higher education level compared to the census [50]. Second, we could not identify the factors that improved the control group’s knowledge. Possibly, they acquired the knowledge during the survey or they were exposed to health promotion strategies elsewhere. However, the results show that behavioral change does not occur merely through improving knowledge. Third, the design was not strictly intention to treat. Using an approach to impute missing outcome data for the relatively large number of dropouts (23.6% of the sample) can cause potential biases. We analyzed them in comparison to their originally assigned group, and confirmed that there were no baseline differences in vegetable intake between the participants included in the analysis and the dropouts. Fourth, regrettably, we have no data on the weight status, health status, or chronic diseases of participants. We did not assess body weight status because the validity of self-assessment of body weight is unknown. Furthermore, a diagnosis by a doctor is necessary to determine the presence or absence of a chronic disease. Exercise is currently being investigated by other project teams; therefore, we could not handle the data on exercise. Other relevant data, such as frequency of intake of other foods, should be examined in the future. This would help in generalizing the results of our study. Fifth, we could not investigate the relationship between the intervention dose and its effect. Regrettably, the website set a common password for all participants because we had insufficient research funding and were unable to handle personal information such as individual ID and password settings. By developing apps or other tools in cooperation with companies in the future, we would be able to further develop this line of research. Finally, the results can only apply at this present time to Japanese individuals aged 30 to 59 years and with incomes less than ¥10,000,000, thus limiting the generalizability of the findings.

### Practical Implications

This program has the following implications. The intervention succeeded in increasing vegetable intake without being restricted to a single geographical area. This shows the possibility that our nutrition education program can spread widely in the future. Furthermore, the program has a systematic composition, containing five steps of four pages of content each: (1) “Today’s point,” 2) “Do you know?” 3) “Easy to devise,” and 4) “Let’s try it!” It is worth investigating whether the program can achieve the same effect using other methods (eg, higher frequency of emails [20 times], face-to-face delivery of content). Further research might aim to clarify which components of Web-based interventions or the program framework contribute to reducing vegetable intake disparities.

### Conclusion

The findings from this RCT indicate that this Web-based nutrition education program can increase vegetable intake among low-income adults, thus contributing to a reduction in vegetable intake disparities.

## Abbreviations

RCT: randomized controlled trial
SES: socioeconomic status
TTM: transtheoretical model

## Appendix

Multimedia Appendix 1 CONSORT‐EHEALTH checklist (V.1.6.1).
